# Role of COX6C and NDUFB3 in septic shock and stroke

**DOI:** 10.1515/med-2024-1050

**Published:** 2024-11-29

**Authors:** Wenbin Tian, Pei Zhang, Ning Yu, Junyu Zhu, Chao Liu, Xuefang Liu, Ya Liu

**Affiliations:** Department of Anesthesiology and Intensive Care, The Second Hospital of Hebei Medical University, Shijiazhuang, China

**Keywords:** COX6C, NDUFB3, septic shock, stroke, bioinformatics

## Abstract

**Background:**

Septic shock is a clinical syndrome characterized by acute circulatory disturbance. Stroke is an acute cerebrovascular disease caused by brain tissue damage. However, the relationship of COX6C and NDUFB3 to them is unclear.

**Method:**

The stroke dataset GSE58294 and the septic shock dataset GSE15491 were downloaded from the gene expression omnibus database. Screening of differentially expressed genes (DEGs), weighted gene co-expression network analysis, construction and analysis of protein–protein interaction network, functional enrichment analysis, gene set enrichment analysis, immune infiltration analysis, and comparative toxicogenomics database (CTD) analysis were performed. Gene expression heat map was drawn. TargetScan screened miRNAs regulating central DEGs.

**Results:**

A total of 664 DEGs were obtained. Gene ontology analysis showed that they were mainly enriched in leukocyte activation, intracellular vesicle, neutrophil activation, and cytokine receptor activity. According to Kyoto Encyclopedia of Genes and Genomes analysis, they are mainly enriched in metabolic pathways, phagosomes, and *Staphylococcus aureus* infection. Core genes (UQCRQ, USMG5 [ATP5MD], COX6C, NDUFB3, ATP5L [ATP5MG], COX7C, NDUFA1, NDUFA4) were highly expressed in septic shock and stroke samples. CTD analysis found that eight core genes are associated with liver enlargement, inflammation, proliferation, fibrosis, and necrosis.

**Conclusion:**

COX6C and NDUFB3 genes are highly expressed in septic shock and stroke. The higher the COX6C and NDUFB3 genes, the worse the prognosis.

## 1 Introduction

Septic shock is a severe systemic inflammatory response syndrome. The incidence of septic shock increases with age, and people over 50 years old are more susceptible. There is no significant difference in incidence between men and women. The mortality rate increases with the degree of multiple organ dysfunction [1–3]. Septic shock is characterized by systemic inflammation, hypotension, multiple organ dysfunction, rapid progression, high mortality rate, and difficult treatment. The condition progresses rapidly with systemic symptoms, with initial symptoms including fever, chills, fatigue, and so on. As the condition progresses, patients may experience symptoms such as altered mental status, tachycardia, and hypotension [4]. The pathological features of septic shock include inflammatory response, coagulation dysfunction, tissue hypoxia, and multiple organ dysfunction [5]. Septic shock is a serious infectious disease that can cause altered mental status, multiple organ dysfunction, bleeding, and infections. It has a high mortality rate. Stroke is a common neurological disorder, also known as a “brain attack.” It occurs when blood supply to the brain is obstructed or ruptured. Stroke is a disease that mainly occurs in people over the age of 60, with no significant difference in incidence between men and women. However, women have a higher incidence at a younger age, while men have a higher incidence in middle and old age. Stroke is more common in Asia and Africa [6–8]. Stroke is characterized by sudden onset, focal involvement, ischemic or hemorrhagic nature, sequelae, high disability, and mortality rates. The clinical manifestations of stroke often include sudden onset of headache, limb paralysis, aphasia, visual impairment, and neurological and psychiatric disorders [9]. Ischemic stroke is caused by the obstruction or narrowing of blood flow in the brain’s blood vessels. Hemorrhagic stroke is caused by the rupture of blood vessels in the brain, leading to cerebral bleeding [10]. Stroke is a serious disease that can cause disability or even death. The exact causes of septic shock and stroke are not fully understood, and it is possible that these diseases may be related to genetic factors, chromosomal abnormalities, gene fusion, and other factors. Therefore, it is particularly important to conduct in-depth research into the molecular mechanisms of septic shock and stroke.

Bioinformatics is an interdisciplinary field that involves computer science, mathematics, biology, and statistics. The development of bioinformatics technology has greatly assisted biological research, accelerating the interpretation and understanding of biomolecules such as genomes, proteins, and metabolomes. Bioinformatics technology includes sequence analysis, structure analysis, functional prediction, systems biology, genomics, and proteomics. With the development of high-throughput sequencing technology and the reduction of costs, a large amount of biological information is stored in public databases. Bioinformatics technology is constantly evolving, allowing for more efficient and accurate interpretation of biological information. The advantages of bioinformatics technology are mainly reflected in its efficiency, accuracy, visualization, and reproducibility.

However, the relationship between COX6C, NDUFB3 genes and septic shock and stroke is currently unclear. This article aims to use bioinformatics techniques to identify the key genes associated with septic shock and stroke compared to normal tissues, and to perform enrichment analysis and pathway analysis. The significant roles of COX6C and NDUFB3 genes in septic shock and stroke will be validated using public datasets.

## 2 Methods

### 2.1 Sepsis shock and stroke dataset

In this study, the configuration files of stroke dataset GSE58294 and sepsis shock dataset GSE154918 were downloaded from the gene expression omnibus (GEO) database (http://www.ncbi.nlm.nih.gov/geo/) generated from GPL570 and GPL20301. GSE58294 includes 69 stroke and 23 normal samples, while GSE154918 includes 19 sepsis shock and 26 normal samples, which were used to identify differentially expressed genes (DEGs) in sepsis shock and stroke.

### 2.2 Screening of DEGs

To ensure the accuracy and reliability of data analysis, the R package “limma” was used to perform probe summarization and background correction on the matrices of GSE58294 and GSE154918. This will ensure high-quality data, providing a solid foundation for subsequent bioinformatics analysis and interpretation of results. The Benjamini–Hochberg method for adjusting raw *p*-values maintains good statistical power, is suitable for large-scale data analysis, and is computationally simple, making it a very practical correction method. The false discovery rate (FDR) was used to calculate fold change (FC). The cut-off value for DEGs was set as *p*-value less than 0.05 and FC greater than 1.5. A volcano plot was created to visually represent the results.

### 2.3 Weighted gene co-expression network analysis (WGCNA)

In this study, the main purpose of calculating the median absolute deviation (MAD) for each gene from the gene expression profile and removing the bottom 50% of genes with the smallest MAD is to reduce noise and instability in the data, aiming to improve the quality of subsequent analyses. Using the R packages WGCNA and Good Samples Genes method to remove outlier genes and samples serves the main purpose of cleaning the data, reducing noise, and mitigating the impact of outliers, thereby enhancing the accuracy and reliability of subsequent data analysis. Constructing co-expression networks were constructed, and we identified gene modules and hub genes. Calculating the correlation between samples, computing gene variability, and identifying outlier samples and genes. To construct a scale-free co-expression network, we calculated the Pearson correlation matrix for all pairs of genes and used the average linkage method to construct a weighted adjacency matrix with a power function *a*_mn = | *c*_mn | ^ *β* (*c*_mn is the Pearson correlation between gene m and gene n and *a*_mn is the adjacency between gene m and gene n), where *β* is a soft-thresholding parameter that emphasizes strong correlations between genes and attenuates weak correlations. After selecting a power of 8, the adjacency was converted to a topological overlap matrix (TOM), which measures the connectivity of genes in the network as the sum of their adjacency with all other genes, and computes the corresponding dissimilarity (1-TOM). To assign genes with similar expression profiles to gene modules, we performed average linkage hierarchical clustering on the gene dendrogram based on the TOM-based dissimilarity measure, with a minimum module size of 30. We set the sensitivity to 3. To further analyze the modules, we calculated the differences between the module feature genes and selected a cut line for the module dendrogram, and merged some modules with a distance less than 0.25. It should be noted that the grey module is considered a set of genes that cannot be assigned to any module.

### 2.4 Construction and analysis of protein–protein interaction (PPI) network

The Search Tool for the Retrieval of Interacting Genes (STRING) database (http://string-db.org/) aims to collect, score, and integrate all publicly available sources of PPI information and supplement these sources with computationally predicted interactions. In this study, the list of DEGs was input into the STRING database to construct a predicted PPI network of core genes (confidence >0.4). Cytoscape software provides powerful network analysis and two-dimensional visualization tools for biologists, helping to understand and explore the complexity of biological systems, thus driving progress in biological research. In this study, we visualized and predicted core genes of the PPI network formed by the string database using Cytoscape software. First, we imported the PPI network into the Cytoscape software, used MCODE to find the best module, and used three algorithms (MCC, MNC, DMNC) to calculate the top genes with the best correlation and took their intersection. The MCC algorithm is used to assess the centrality and importance of nodes in a network by identifying the maximum cliques (largest complete subgraphs) in the network. The MNC algorithm evaluates the importance of nodes in their neighborhood components by identifying the maximum neighborhood component of each node. The DMNC algorithm assesses the density and relationship between nodes and their neighborhood components. It considers not only the connectivity of nodes but also the density of neighborhood components. By using a combination of the MCODE algorithm and these three algorithms, important gene modules can be effectively identified in biological networks. Subsequently, highly correlated core genes can be selected for further analysis, thereby advancing biological research. Finally, we visualized and exported the core gene list.

### 2.5 Functional enrichment analysis

Using Gene ontology (GO) and Kyoto Encyclopedia of Genes and Genomes (KEGG) for analysis helps to understand the functions and interactions of genes and proteins, revealing the regulatory mechanisms and signaling pathways of biological systems. This provides important references and guidance for biological research. In this study, the DEG list selected by the Venn diagram was inputted into the KEGG API (https://www.kegg.jp/kegg/rest/keggapi.html) to obtain the latest gene annotation for KEGG pathway, which was used as the background. The genes were mapped to the background set, and clusterProfiler (version 3.14.3) R package was used to perform enrichment analysis to obtain the enrichment results of the gene set. Hypergeometric test, multiple hypothesis testing correction, result interpretation, and visualization: through these statistical analysis steps, the clusterProfiler R package can provide rich enrichment results for gene sets, helping researchers to gain insight into the functional characteristics, pathway involvement, and relevant biological processes of the gene set. The GO annotation of genes in org.Hs.eg.db (version 3.1.0) R package was also used as the background. The genes were mapped to the background set, and a minimum gene set of 5 and a maximum gene set of 5,000 were set. A *p-*value of <0.05 and a FDR of < 0.25 were considered as statistically significant criteria.

In addition, the Metascape database can provide comprehensive gene list annotation and analysis resources, as well as visualization export. We used the Metascape (http://metascape.org/gp/index.html) database to perform functional enrichment analysis and export for the differential gene list mentioned above.

### 2.6 Gene set enrichment analysis (GSEA)

GSEA can more effectively uncover the biological significance of gene expression data, identify related functional pathways and biological processes, and thus provide deeper insights and guidance for research. For GSEA, we obtained the GSEA software (version 3.0) from the GSEA website (DOI: 10.1073/pnas.0506580102, http://software.broadinstitute.org/gsea/index.jsp). Samples were divided into two groups based on sepsis shock and normal samples, as well as stroke and normal samples, and the c2.cp.kegg.v7.4.symbols.gmt subset was downloaded from the Molecular Signatures Database (DOI: 10.1093/bioinformatics/btr260, http://www.gsea-msigdb.org/gsea/downloads.jsp) to evaluate the relevant pathways and molecular mechanisms. Based on gene expression profiles and phenotype grouping, the minimum gene set was set to 5, the maximum gene set was set to 5,000, and 1,000 resamplings were performed. A *p-*value of <0.05 and a FDR of <0.25 were considered statistically significant. Additionally, GO and KEGG analysis were performed on the entire genome. The GSEA software was used to carry out these analyses.

### 2.7 Gene expression heatmap

We used the R package heatmap to generate heatmaps of the expression levels of the core genes identified by the three algorithms in the PPI network in GSE58294 and GSE154918, visualizing the expression differences of the core genes between stroke and normal samples and between septic shock and normal samples.

### 2.8 Immune infiltration analysis

CIBERSORT (http://CIBERSORT.stanford.edu/) is a widely used computational method for calculating immune cell infiltration. CIBERSORT provides a powerful tool for deciphering the cellular composition of complex tissues, revealing cell–cell interactions and the dynamic changes of cells within biological processes. The LM22 gene signature is used to define 22 immune cell subtypes. In this study, we applied an integrated bioinformatics approach and used the CIBERSORT package to analyze the gene expression profiles of GSE58294 and GSE154918. We utilized the principle of linear support vector regression to deconvolute the expression matrix of immune cell subtypes and estimate the abundance of immune cells. We also selected samples with sufficient confidence with a cut-off of *p* < 0.05.

### 2.9 Comparative toxicogenomics database (CTD) analysis

The CTD integrates vast amounts of data on interactions between chemical substances, genes, functional phenotypes, and diseases, providing great convenience for research on environmental exposure factors and potential mechanisms of drug action related to diseases. We input the core genes into the CTD website, found the most relevant diseases with the core genes, and used Excel to draw pie charts of the expression differences of each gene.

### 2.10 miRNA

DIANA-TarBase (dianalab.e-ce.uth.gr) is a bioinformatics tool used for retrieving and validating miRNA target gene information. It is based on a large amount of experimental data and provides extensive information on the interactions between miRNAs and their target genes. Key statistical analyses include methods of experimental validation, literature support, and functional annotation of target genes. Through these statistical analyses, DIANA-TarBase can provide reliable relationships between miRNAs and target genes and includes experimental validation data to help identify and confirm functional miRNA target genes. These analyses are crucial for understanding the role of miRNAs in gene regulatory networks and their functions in diseases and biological processes. In this study, we utilized DIANA-TarBase to screen miRNAs regulating DEGs in our regulatory network analysis.


**Ethical approval:** The data in this article are from public databases and are exempt from ethical review.

## 3 Result

### 3.1 Differential expression gene analysis

**Figure 1:**
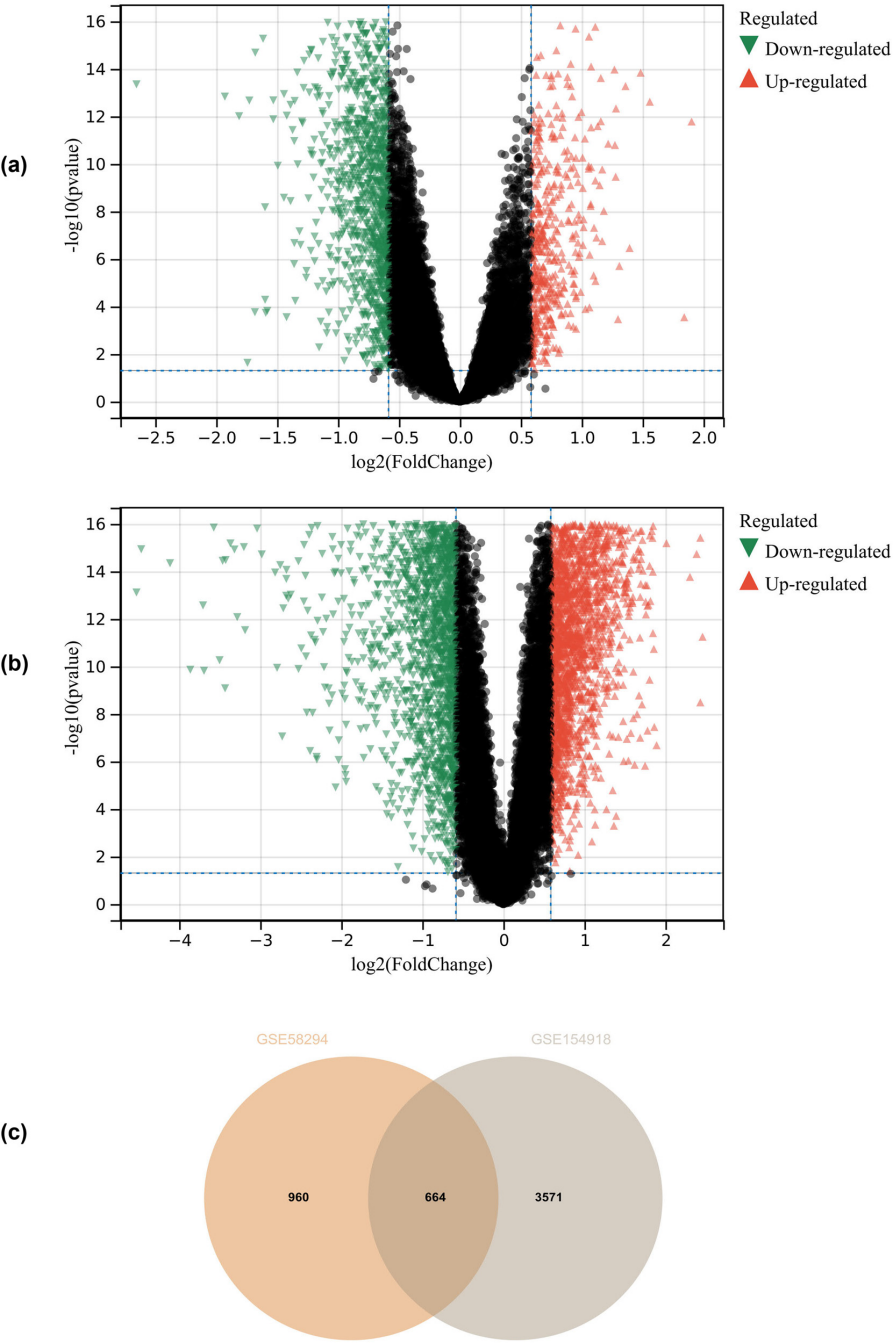
Differential expression gene analysis: (a) GSE58294, (b) GSE154918, and (c) a total of 664 DEGs.

In this study, using the pre-defined cut-off values, DEGs were identified from the GSE58294 and GSE154918 matrices (Figure 1a for GSE58294 and Figure 1b for GSE154918), resulting in a total of 664 DEGs (Figure 1c).

### 3.2 Functional enrichment analysis

#### 3.2.1 DEGs

**Figure 2:**
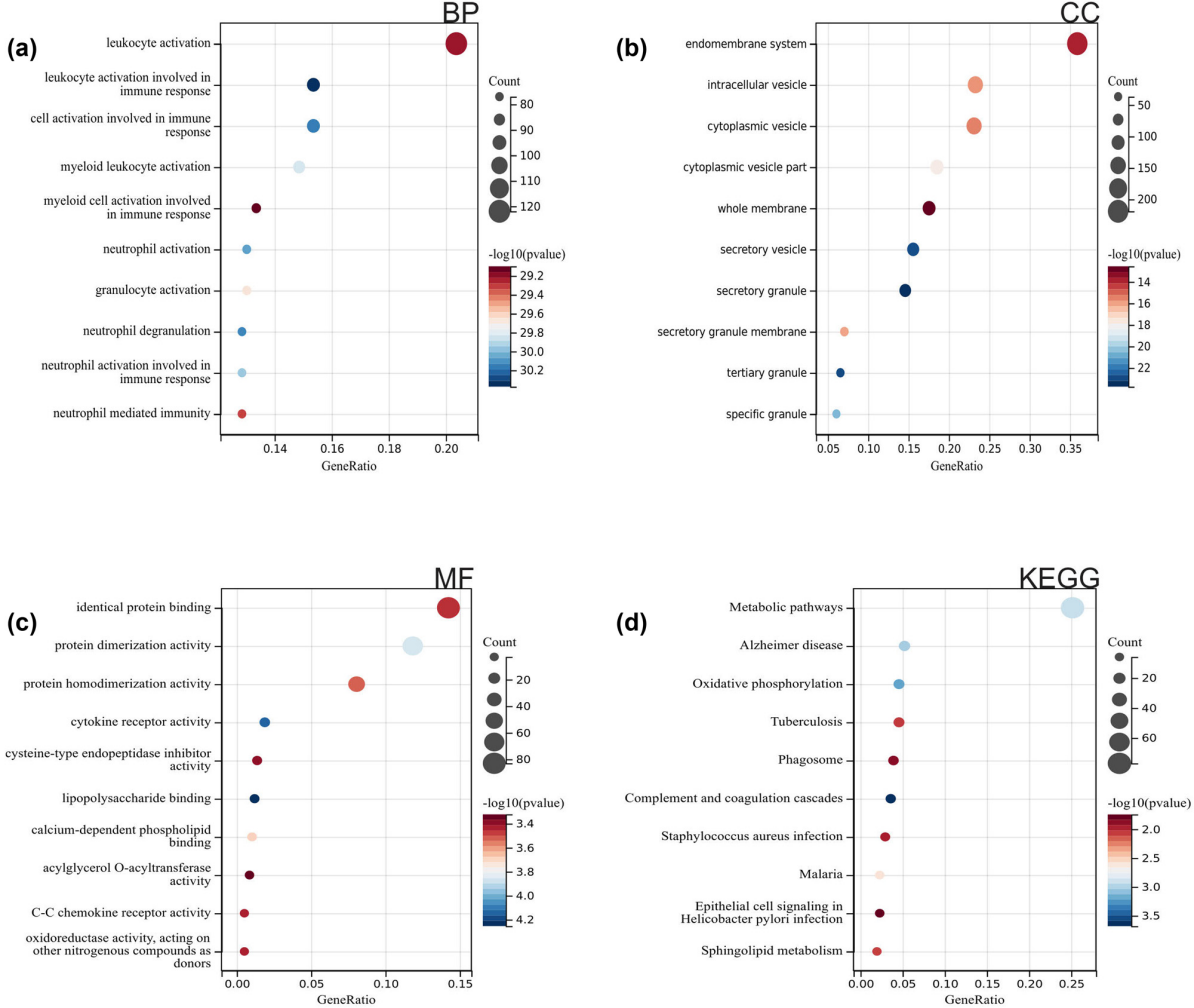
Functional enrichment analysis: (a)–(c) GO and (d) KEGG analysis.

We performed GO analysis on these DEGs, and according to the results, they were mainly enriched in leukocyte activation, intracellular vesicle, neutrophil activation, and cytokine receptor activity (Figure 2a–c). According to the KEGG analysis, they are mainly enriched in metabolic pathways, phagosomes, and *Staphylococcus aureus* infection (Figure 2d).

#### 3.2.2 GSEA

**Figure 3:**
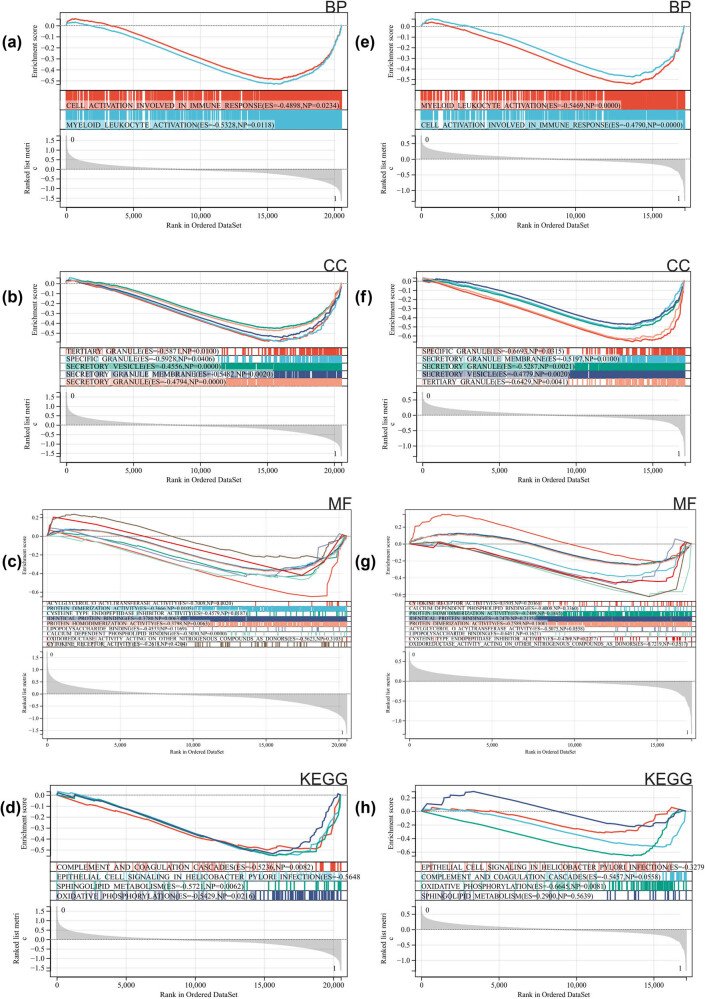
GSEA: (a)–(d) GSE58294 and (e)–(h) GSE154918.

According to our GSEA enrichment analysis of the whole genome, we aimed to identify enrichment projects that may exist in non-DEGs and validate the results of DEGs. The intersection of the enrichment items with the GO and KEGG enrichment items of the DEGs. They are mainly enriched in myeloid cell activation, cytokine receptor activity, and oxidative phosphorylation (Figure 3a–d for GSE58294 results and Figure 3e–h for GSE154918 results).

#### 3.2.3 Metascape enrichment analysis

**Figure 4:**
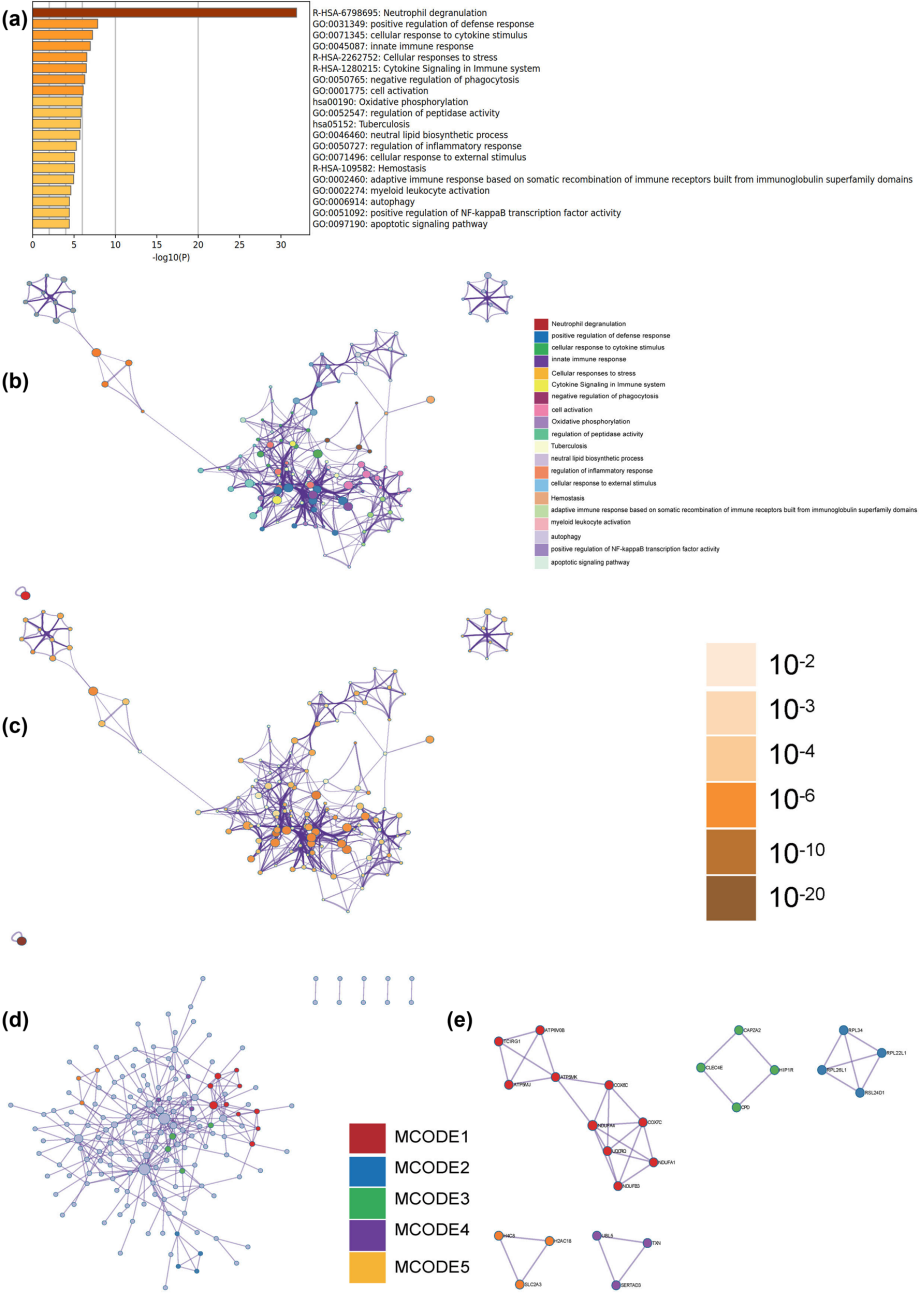
Metascape enrichment analysis. (a) In the Metascape enrichment analysis, we observed enrichment of apoptotic signaling pathway, response to cytokine stimulus, and cell activation in the GO enrichment category. (b)–(e) Enrichment networks colored by enrichment and *p*-value.

In the Metascape enrichment analysis, we observed enrichment of apoptotic signaling pathway, response to cytokine stimulus, and cell activation in the GO enrichment category (Figure 4a). We also generated enrichment networks colored by enrichment and *p*-value (Figure 4b–e), which visualize the associations and confidence of each enrichment item.

### 3.3 WGCNA

**Figure 5:**
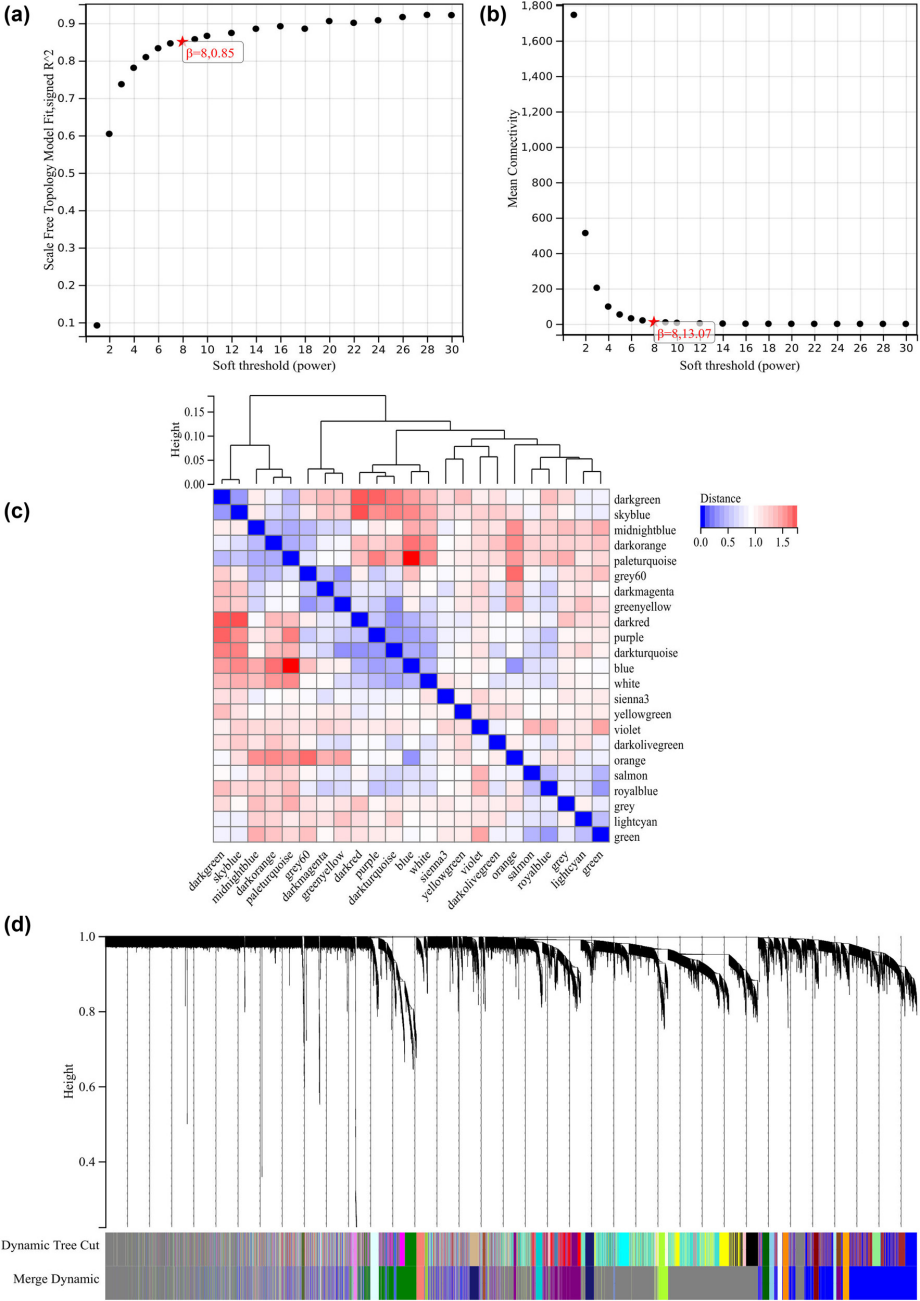
WGCNA: (a) *β* = 80.85 and (b) *β* = 813.07. (c) and (d) Hierarchical clustering trees of all genes were constructed, and important modules were generated, followed by analysis of the interactions between these modules.

**Figure 6:**
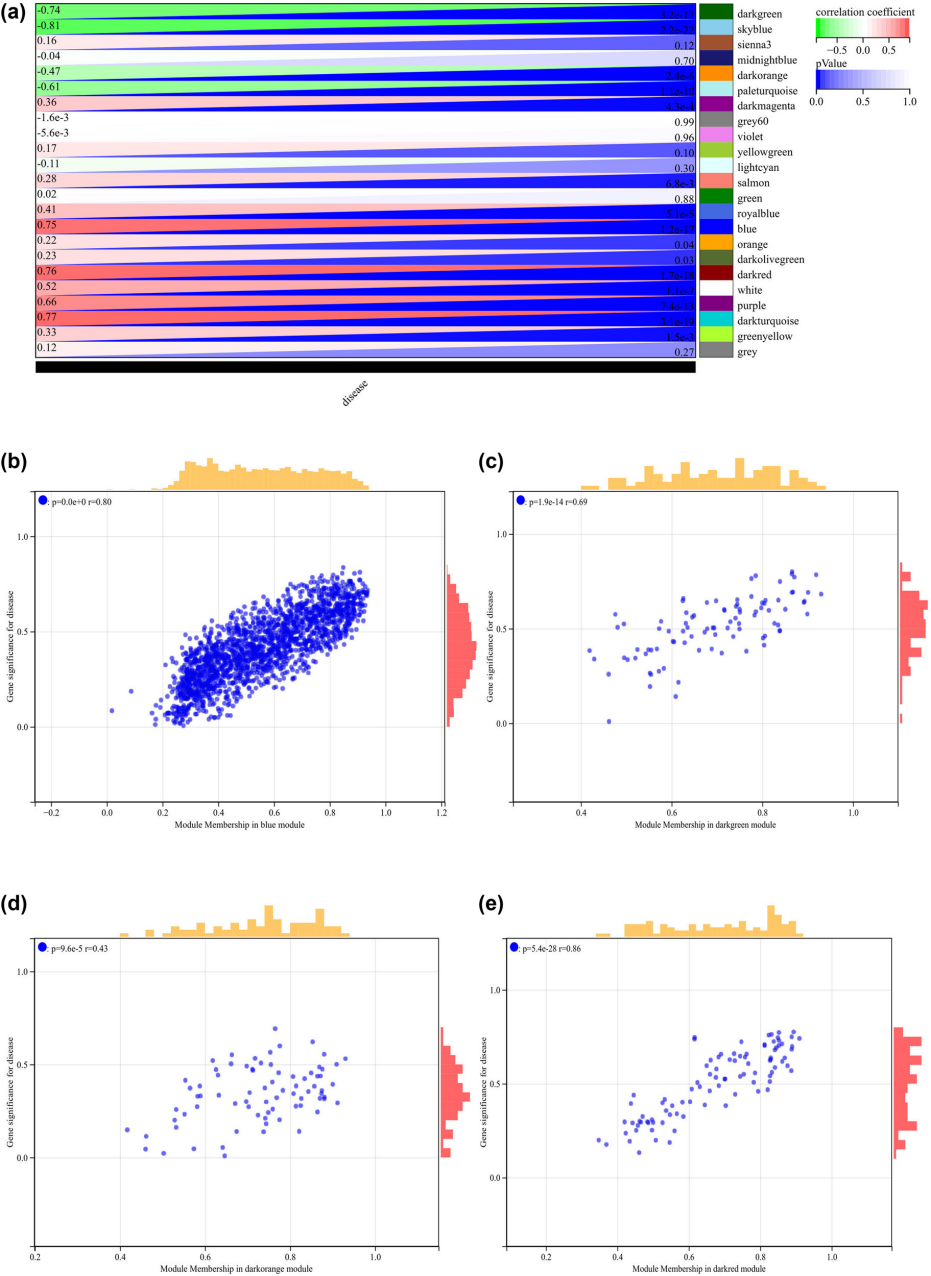
WGCNA: (a) heatmap of module–trait correlations and (b)–(e) the scatterplots of GS versus MM correlations for the related hub genes.

**Figure 7:**
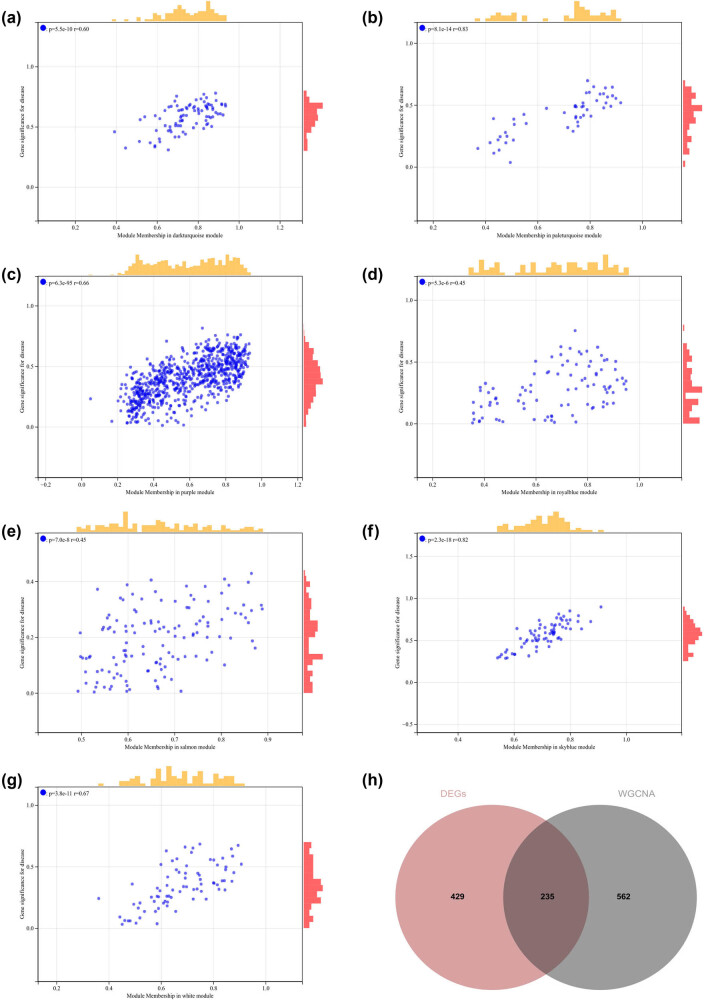
WGCNA: (a)–(g) the scatterplots of GS versus MM correlations for the related hub genes and (h) Venn diagram of WGCNA and DEGs to obtain the intersection of 235 genes.

The selection of soft-thresholding power is an important step in WGCNA. Network topology analysis was performed to determine the soft-thresholding power, which was set to 8 (Figure 5a and b). Hierarchical clustering trees of all genes were constructed, and important modules were generated, followed by analysis of the interactions between these modules (Figure 5c and d). A heatmap of module–trait correlations was generated (Figure 6a), as well as scatterplots of GS versus module member (MM) correlations for the related hub genes (Figures 6b–e and 7a–g).

We calculated the module eigengene–gene expression correlation to obtain MM. According to the cut-off criteria (|MM| > 0.8), 798 highly connected genes in clinically significant modules were identified as hub genes.

We also drew a Venn diagram of WGCNA and DEGs to obtain the intersection of 235 genes, which were used to create and analyze the PPI network (Figure 7h).

### 3.4 Construction and analysis of PPI network

**Figure 8:**
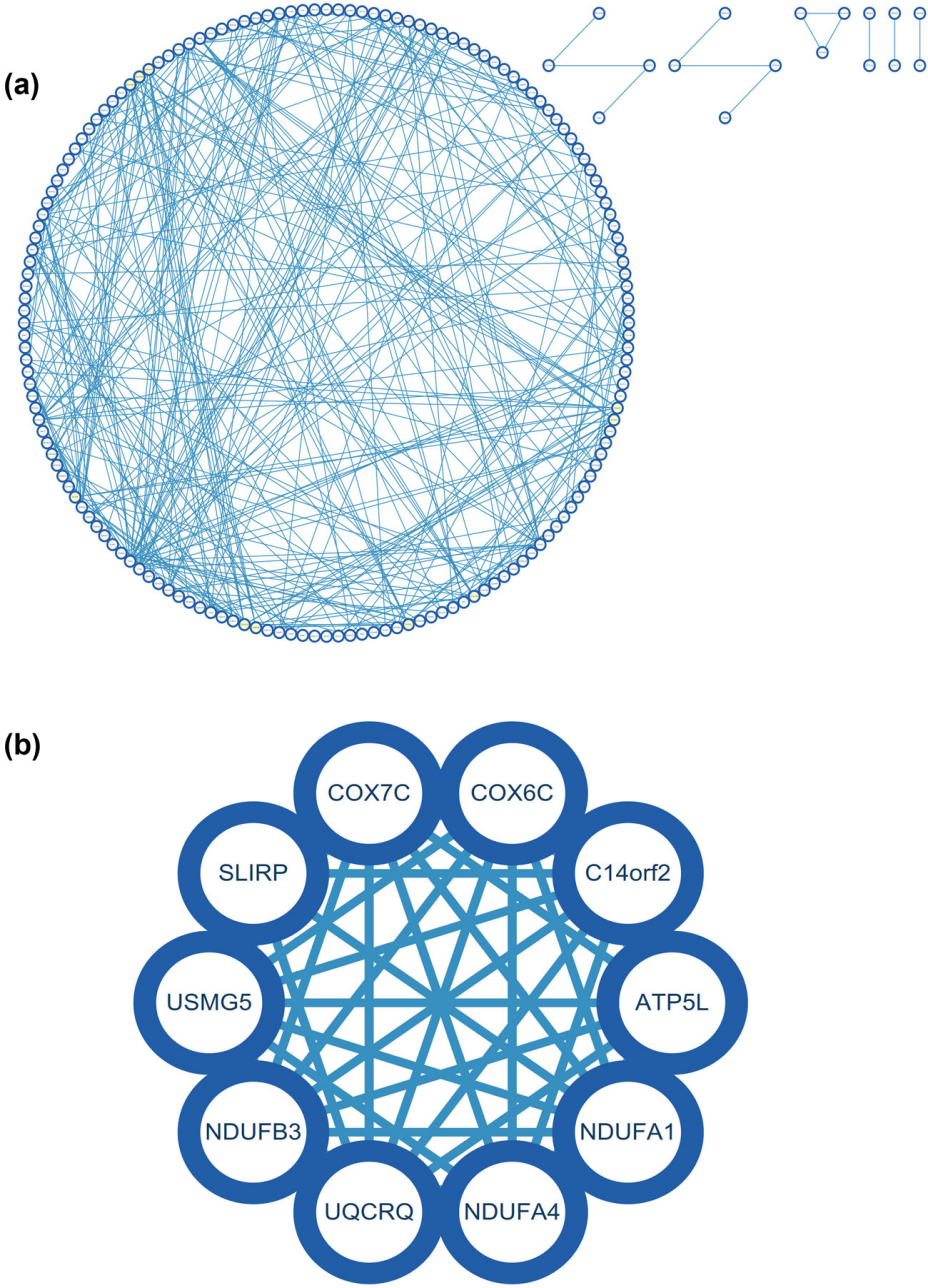
Construction and analysis of PPI network: (a) PPI network of DEGs and (b) core gene clusters.

**Figure 9:**
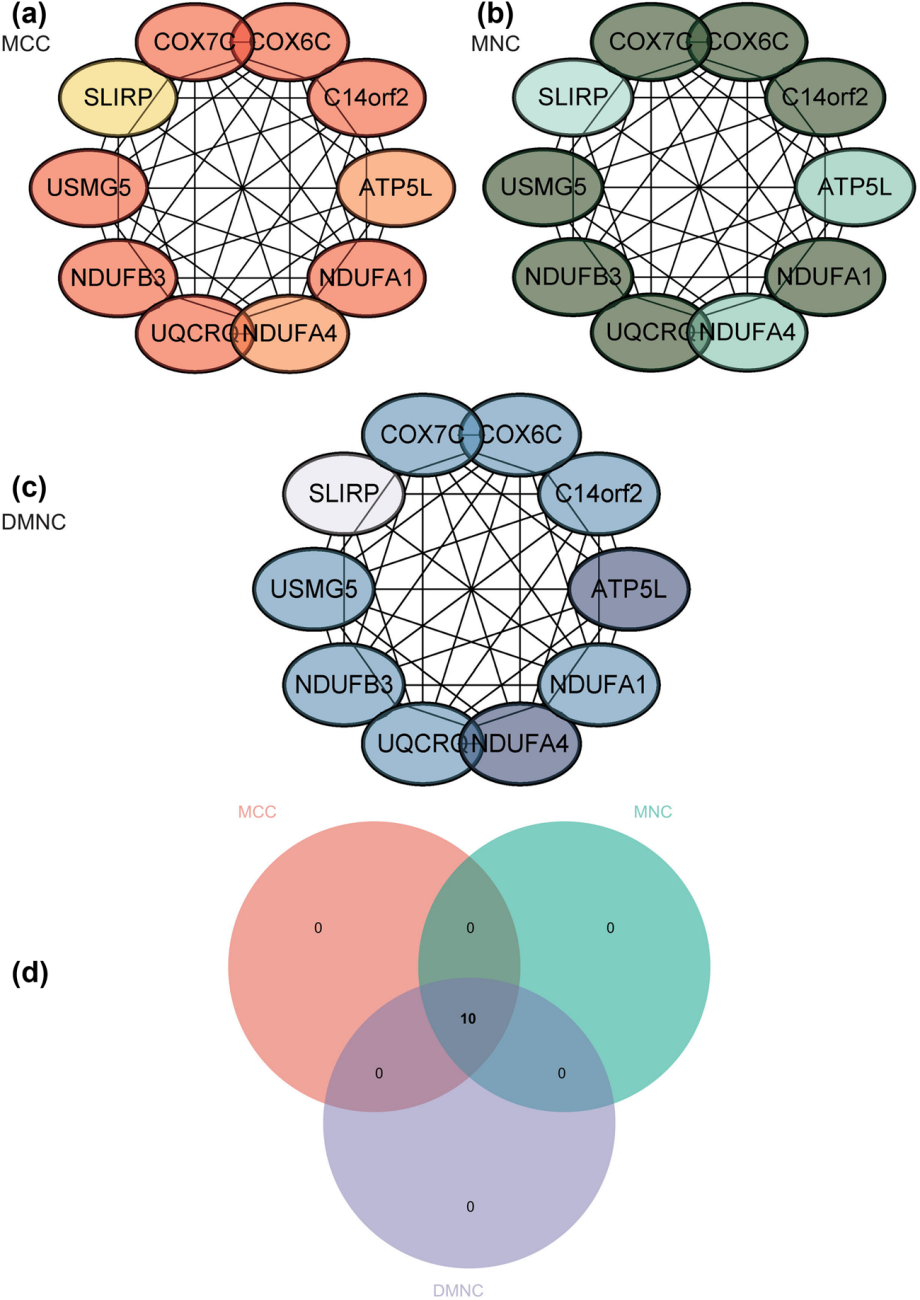
Construction and analysis of PPI network. Three different algorithms were used to identify central genes of (a) MCC, (b) MNC, (c) DMNC. (d) The intersection was obtained using a Venn diagram.

It was constructed and analyzed by Cytoscape software using the PPI network of DEGs obtained from the STRING online database (Figure 8a), and core gene clusters were identified (Figure 8b). Three different algorithms were used to identify central genes (Figure 9a–c), and the intersection was obtained using a Venn diagram (Figure 9d), resulting in ten core genes (COX7C, UQCRQ, COX6C, NDUFB3, USMG5 [ATP5MD], C14orf2, NDUFA1, NDUFA4, ATP5L [ATP5MG], SLIRP).

### 3.5 Gene expression heatmap

**Figure 10:**
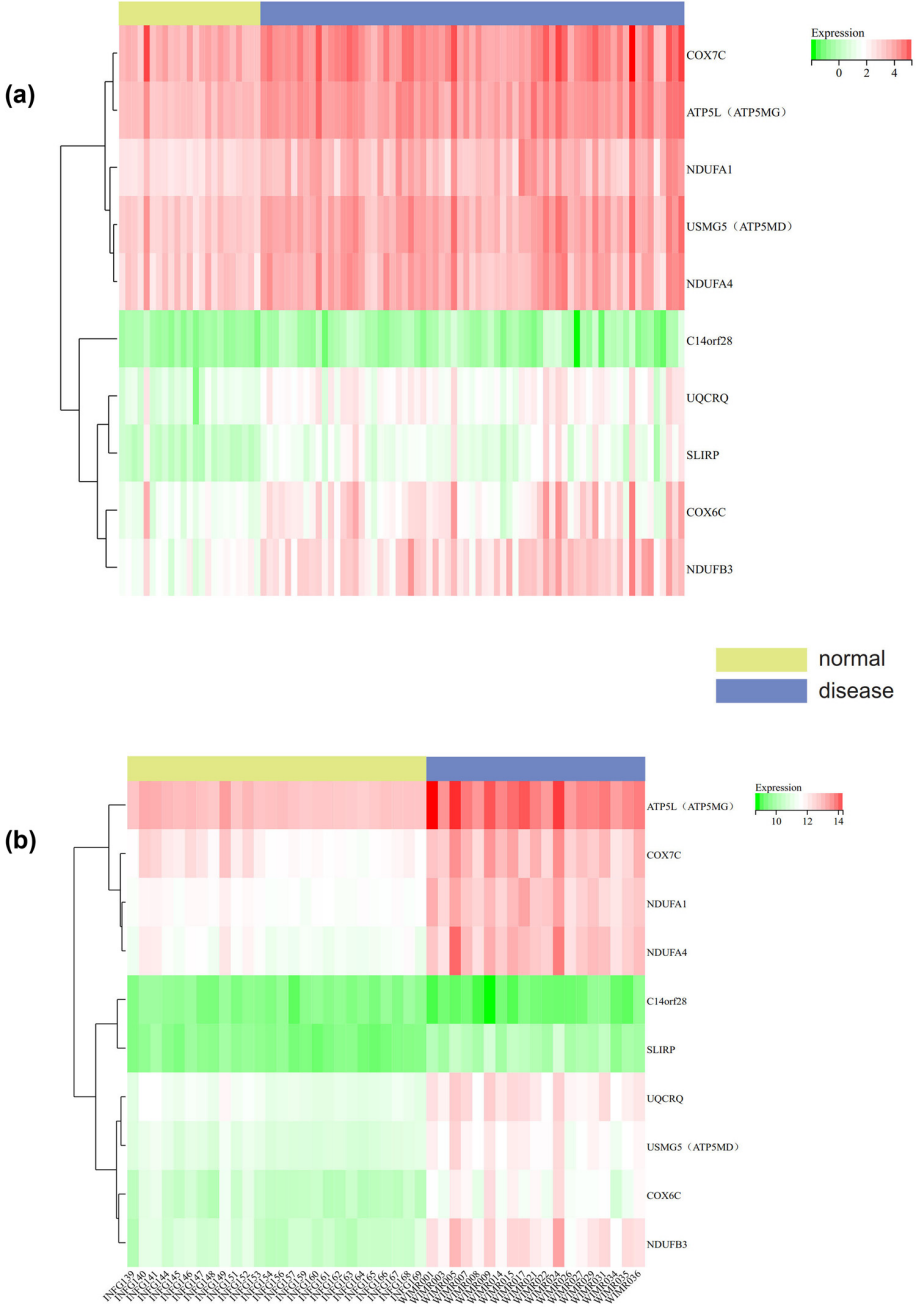
Gene expression heatmap: (a) GSE58294 and (b) GSE154918.

We visualized the heatmap of the expression levels of core genes in the samples (Figure 10a for GSE58294 and Figure 10b for GSE154918), and found that the core genes (UQCRQ, USMG5 [ATP5MD], COX6C, NDUFB3, ATP5L [ATP5MG], COX7C, NDUFA1, NDUFA4) were highly expressed in septic shock and stroke samples, while lowly expressed in normal samples.

### 3.6 CTD analysis

**Figure 11:**
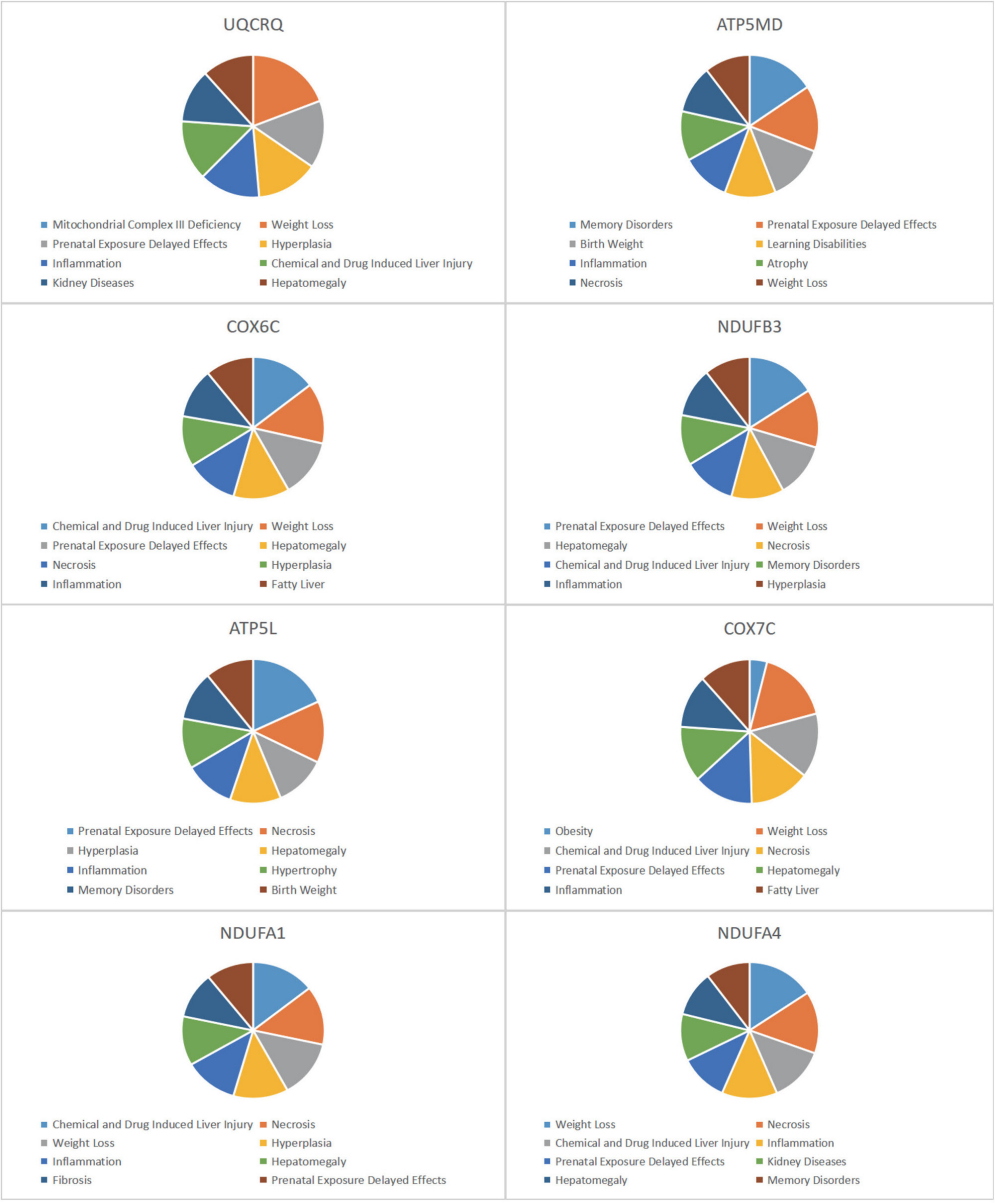
CTD analysis. Eight genes (UQCRQ, USMG5 (ATP5MD), COX6C, NDUFB3, ATP5L [ATP5MG], COX7C, NDUFA1, NDUFA4) are associated with liver enlargement, inflammation, proliferation, fibrosis, and necrosis.

In this study, we input the list of core genes into the CTD website to search for diseases related to these genes, improving our understanding of the association between genes and diseases. We found that eight genes (UQCRQ, USMG5 [ATP5MD], COX6C, NDUFB3, ATP5L [ATP5MG], COX7C, NDUFA1, NDUFA4) are associated with liver enlargement, inflammation, proliferation, fibrosis, and necrosis (Figure 11).

### 3.7 Immune infiltration analysis

**Figure 12:**
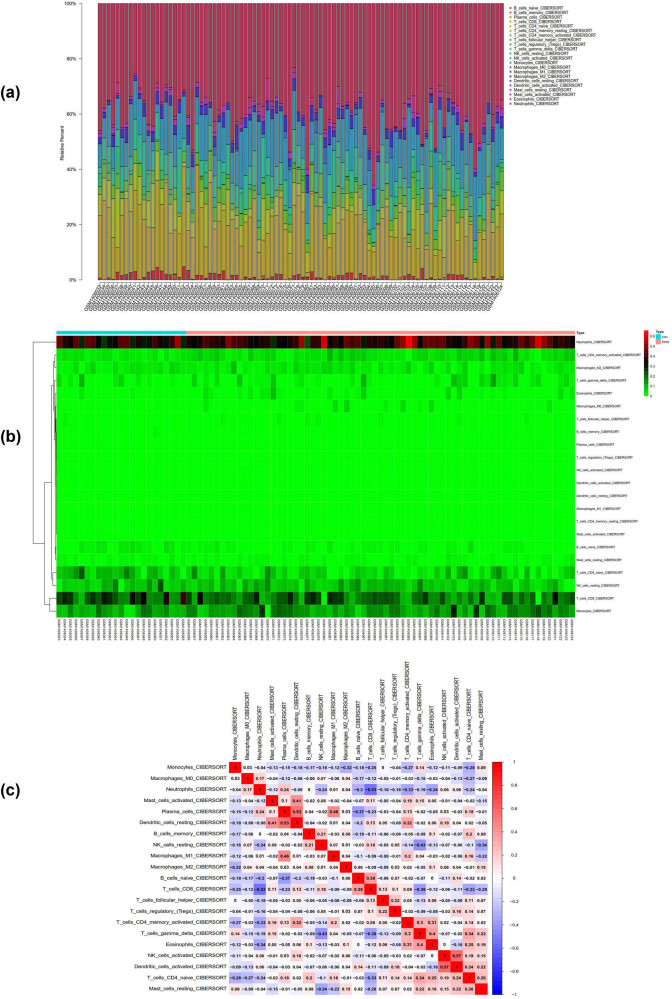
Immune infiltration analysis of GSE58294. (a) The proportions of immune cells in the whole gene expression matrix. (b) A heatmap of immune cell expression in the dataset. (c) The co-expression pattern map of immune cell components.

We performed analysis of the GSE58294 matrix using the CIBERSORT package, and obtained the proportions of immune cells in the whole gene expression matrix at a 95% confidence level (Figure 12a), as well as a heatmap of immune cell expression in the dataset (Figure 12b). We also conducted co-expression correlation analysis of infiltrating immune cells, and obtained a co-expression pattern map of immune cell components (Figure 12c).

**Figure 13:**
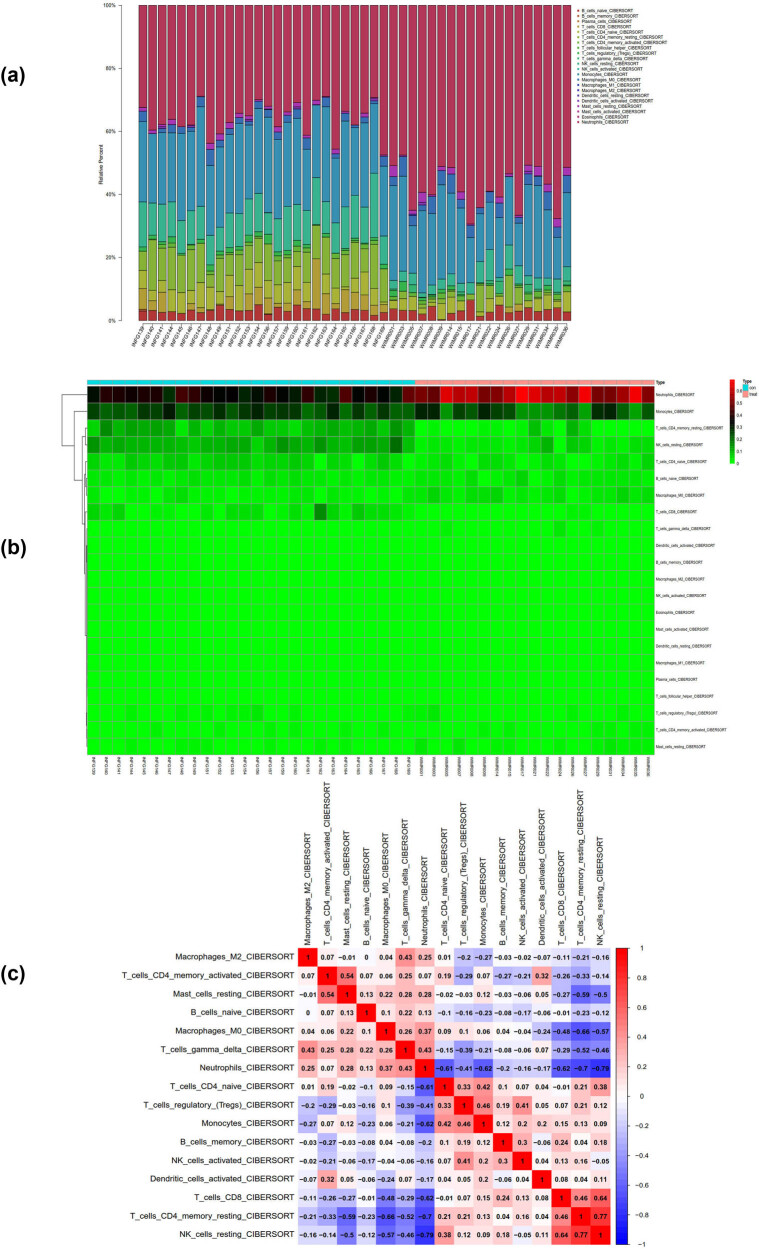
Immune infiltration analysis of GSE154918. (a) The proportions of immune cells in the whole gene expression matrix. (b) A heatmap of immune cell expression in the dataset. (c) The co-expression pattern map of immune cell components.

In this study, we also analyzed the matrix of GSE154918 using the CIBERSORT package. At a 95% confidence interval, we obtained the proportions of immune cells in the whole gene expression matrix (Figure 13a) and a heatmap of immune cell expression in the dataset (Figure 13b). We also performed co-expression correlation analysis of infiltrating immune cells and obtained a co-expression pattern map of immune cell components (Figure 13c).

### 3.8 Prediction and functional annotation of miRNAs related to hub genes

In this study, we inputted the hub gene list into targetScan to search for related miRNAs, which improved our understanding of gene expression regulation (Table 1). We found that the miRNAs associated for the COX6C gene are hsa-miR-221-3p, hsa-miR-19b-3p, and hsa-miR-19a-3p; for the NDUFB3 gene are hsa-miR-543, hsa-miR-26b-5p, and hsa-miR-335-3p; for the UQCRQ gene are hsa-miR-7-5p, hsa-miR-196a-5p, and hsa-miR-29b-3p; for the USMG5 (ATP5MD) gene are hsa-miR-301a-3p, hsa-miR-454-3p, and hsa-miR-130a-3p; for the ATP5L (ATP5MG) gene are hsa-miR-24-3p, hsa-miR-17-5p, and hsa-miR-484; for the COX7C gene are hsa-miR-191-5p, hsa-miR-17-5p, and hsa-miR-34a-5p; for the NDUFA1 gene are hsa-miR-15b-3p, hsa-miR-7-5p, and hsa-miR-92a-3p; and for the NDUFA4 gene are hsa-miR-7-5p, hsa-miR-34a-5p, and hsa-miR-424-5p.

**Table 1:** Summary of miRNAs that regulate hub genes

Gene	miRNA		
COX6C	hsa-miR-221-3p	hsa-miR-19b-3p	hsa-miR-19a-3p
NDUFB3	hsa-miR-543	hsa-miR-26b-5p	hsa-miR-335-3p
UQCRQ	hsa-miR-7-5p	hsa-miR-196a-5p	hsa-miR-29b-3p
USMG5 (ATP5MD)	hsa-miR-301a-3p	hsa-miR-454-3p	hsa-miR-130a-3p
ATP5L (ATP5MG)	hsa-miR-24-3p	hsa-miR-17-5p	hsa-miR-484
COX7C	hsa-miR-191-5p	hsa-miR-17-5p	hsa-miR-34a-5p
NDUFA1	hsa-miR-15b-3p	hsa-miR-7-5p	hsa-miR-92a-3p
NDUFA4	hsa-miR-7-5p	hsa-miR-34a-5p	hsa-miR-424-5p

## 4 Discussion

Sepsis shock is a severe complication of infection, usually caused by bacterial infections. Its molecular mechanisms involve multiple pathophysiological processes, including activation and dysregulation of the immune system, release of inflammatory mediators, and cellular damage, among others. Bacterial infections can activate the immune system, triggering an inflammatory response. Immune cells such as macrophages and dendritic cells in the immune system recognize and engulf bacteria to initiate an immune response. Inflammatory mediators such as tumor necrosis factor-alpha, interleukin-1, and interleukin-6 are released during the inflammatory response, leading to vasodilation and tissue damage [11–13]. The release of inflammatory mediators can cause an increase in microvascular permeability, leading to the leakage of fluid from blood vessels into the tissue space, resulting in tissue edema [14,15]. Inflammatory response may also lead to thrombosis and microcirculatory dysfunction [16,17]. The release of inflammatory mediators and vasodilation may also lead to cellular injury and apoptosis, particularly in important cell types such as endothelial cells and cardiomyocytes. Stroke refers to a disease caused by cerebral vascular disease, which leads to inadequate blood supply or bleeding in the brain, resulting in brain tissue damage and functional impairment. Its molecular mechanisms involve various pathophysiological processes, including vascular changes, inflammatory response, and neuronal cell death. Vascular changes may include arteriosclerosis, stenosis, thrombosis, and vascular rupture. Inflammatory response can cause brain cell damage and neuroinflammation by releasing a series of inflammatory mediators such as interleukins, tumor necrosis factor, and interferons [18–20]. Neuronal cell death may be caused by various mechanisms such as intracellular energy metabolism disorders, cell membrane damage, and apoptosis. Neuronal cell death can lead to impaired brain function [21,22]. Ischemia and hypoxia can also lead to the generation of free radicals, which can combine with lipids and proteins, causing oxidative damage [23,24]. After a stroke, the plasticity of neurons changes, including changes in synaptic plasticity and neuronal composition. These changes in plasticity may lead to either recovery or deterioration of brain function. A deeper understanding of the molecular mechanisms underlying septic shock and stroke is crucial for the development of targeted drugs. The main finding of this study is that the COX6C and NDUFB3 genes are highly expressed in septic shock and stroke, and the higher the expression of these genes, the worse the prognosis.

Shock and stroke are two severe diseases related to the circulatory system, with their pathogenesis involving complex genetic and environmental factors [25]. The onset of shock may be associated with variations in immune response genes. Studies have shown that polymorphisms in specific immune regulatory genes are linked to susceptibility to septic shock [26]. In recent years, through genome-wide association studies and other genetic methods, candidate genes and genomic regions related to shock have been identified, including genes associated with immune regulation, inflammatory response, and microcirculation [27]. Shock is often accompanied by the activation of inflammatory responses, and variations in certain inflammation-related genes may affect the development and severity of shock. Metabolic pathway disorders are also associated with shock, and gene polymorphisms involved in metabolic processes may be related to the susceptibility and prognosis of shock. Vascular dysfunction plays an important role in the development of shock, and genetic variations affecting vascular tone and hemodynamics may lead to the progression of shock. Stroke can be a manifestation of some hereditary diseases, such as familial cerebral aneurysms and familial hypercholesterolemia. These diseases have clear genetic patterns and are closely related to the occurrence of stroke. Large-scale genetic epidemiological studies have shown that stroke risk is influenced by multiple genetic variations, including genes related to vascular health, thrombosis, and cerebral blood flow regulation. Genetic loci associated with stroke risk may involve various biological processes, including the development of atherosclerosis, platelet aggregation, and thrombus formation. Thrombosis is a major mechanism of stroke. Gene polymorphisms may affect the processes of thrombus formation and dissolution. Vascular health is crucial for the onset and prognosis of stroke. Variations in genes related to vascular wall structure and function (such as COL3A1, EDN1) may influence stroke risk. Inflammatory responses and immune function are also associated with stroke onset. Variations in genes such as IL-6 and TNF-α may affect inflammatory responses and stroke development. During a stroke, abnormalities in cerebral blood flow regulation are a critical factor. Gene polymorphisms may affect the autoregulation of cerebral blood vessels, thus influencing the occurrence and prognosis of stroke. Genetic research on shock and stroke involves multiple aspects, including immune response, inflammatory response, metabolic pathways, vascular function, and thrombosis.

COX6C is a human gene that encodes a subunit of the mitochondrial cytochrome c oxidase (COX) complex. COX is an important component of respiratory chain complex IV, and it participates in the final step of the respiratory chain process in mitochondria, converting electrons and oxygen into water while releasing energy. COX6C is one of the subunits of the COX complex, and it works together with other subunits to form a catalytically active complex. The expression of COX6C is closely related to oxidative phosphorylation processes in mitochondria, and it may play an important role in regulating energy metabolism and cellular respiration in mitochondria [28]. NDUFB3 is a human gene that encodes a protein which is one of the subunits of mitochondrial respiratory chain complex I. Mitochondrial respiratory chain complex I is the first and largest complex in the mitochondrial respiratory chain, and it is involved in transferring electrons from substrates such as NADH and FADH2 to other complexes in the mitochondrial respiratory chain, while generating energy. NDUFB3, as a subunit of complex I, is involved in the assembly and stability of the complex, and also has some influence on the catalytic activity of complex I [29]. Studies have suggested that COX6C may be involved in oxidative stress caused by mitochondrial dysfunction [30]. The reactive OXPHOS of NDUFB3 is correlated with ATP levels [31]. It is therefore speculated that the COX6C and NDUFB3 genes may play an important role in the inflammatory response, oxidative stress, and metabolic disorders in septic shock. Another study has shown that COX6C involvement in cerebral ischemia proteins may allow the identification of putative biomarkers or therapeutic targets for ischemic stroke [32]. We observed the upregulation of various components of the OxPhos machinery including NDUFB3, which led to the generation of mitochondrial ROS, promoting the formation and activation of NLRP3 inflammasome, and subsequently pyroptosis [33]. Therefore, it is speculated that COX6C and NDUFB3 genes play an important role in the process of ischemia–hypoxia, neuronal apoptosis, and neuroinflammatory response in stroke.

Although this article has conducted rigorous bioinformatics analysis, there are still some shortcomings. This study did not conduct animal experiments on gene overexpression or knockout to further validate its function. Therefore, in future research, we should conduct in-depth exploration in this area.

## 5 Conclusion

COX6C and NDUFB3 are highly expressed in septic shock and stroke, and may play a significant role in the development of septic shock and stroke through cellular regulation and other pathways. COX6C and NDUFB3 may serve as molecular targets for precise treatment of septic shock and stroke, providing a certain direction for the mechanism research of septic shock and stroke.

## Abbreviations

CTD: comparative toxicogenomics database
DEGs: differentially expressed genes
FC: fold change
FDR: false discovery rate
GEO: gene expression omnibus
GO: gene ontology
GSEA: gene set enrichment analysis
KEGG: Kyoto encyclopedia of genes and genomes
MAD: median absolute deviation
MM: module membership
PPI: protein–protein interaction
STRING: search tool for the retrieval of interacting genes
WGCNA: weighted gene co-expression network analysis
